# *Mustn1* ablation in male mice results in fiber type and gene expression alterations during skeletal muscle regeneration

**DOI:** 10.14814/phy2.70772

**Published:** 2026-02-19

**Authors:** Kaitlin Tagliaferri, Nithya Thomas, Michael Atallah, Stavroula Triantafillou, Michael Hadjiargyrou

**Affiliations:** ^1^ College of Osteopathic Medicine New York Institute of Technology Old Westbury New York USA; ^2^ Department of Biological & Chemical Sciences New York Institute of Technology Old Westbury New York USA

**Keywords:** cardiotoxin, fiber‐type, Mustn1, regeneration, skeletal muscle

## Abstract

We previously developed a Mustn1 conditional knockout (KO) mouse model targeting Pax7‐expressing skeletal muscle satellite cells and showed its role in glucose metabolism, strength, gait, peak contractile strength, and myofiber composition. To investigate Mustn1's role in muscle regeneration, we used these KO mice in a cardiotoxin (CTX)‐induced tibialis anterior injury model. Despite no major histological differences or deficits in ladder climbing between KO and wild‐type (WT) mice at post‐injury (Day 2–10), we observed significant shifts in fiber type composition. Mustn1 KO mice had more Type IIa fibers at Day 5, while Type IIx and IIb fibers were reduced at Day 2 and 10, respectively. Additionally, we observed increases in Type I fiber cross‐sectional area in the Mustn1 KO mice at Day 0 and 2. Lower numbers of centrally nucleated fibers were also seen in the Mustn1 KO mice at Day 10. Pax7^+^ cells were also greater in numbers in the Mustn1 KO mice at Day 2 and 10. Lastly, expression of myogenic genes also differed significantly between the two strains. These data suggest that Mustn1 is integral to skeletal muscle fiber composition and myogenic gene expression thereby facilitating muscle repair and regeneration.

## 1 INTRODUCTION

Skeletal muscle is a dynamic tissue capable of regeneration following injury, relying on a highly orchestrated process involving muscle stem cells, extracellular matrix composition, as well as regulatory and structural proteins (Li et al., 2025). Central to this process are satellite cells, quiescent muscle progenitors, marked by Pax7, which become activated upon injury to facilitate muscle repair (Schmidt et al., 2019). Effective regeneration depends on the precise coordination of myogenic differentiation markers (e.g., MyoD, MyoG), structural proteins (e.g., Desmin, Myh4), and cell adhesion/fusion molecules (e.g., Cadh, Cap1, Cav3) (Mukund & Subramaniam, 2020). Although, many regulatory mechanisms governing these processes are understood, there are others that remain unknown.

Mustn1 (Musculoskeletal, Embryonic Nuclear Protein 1) has emerged as a critical gene involved in skeletal muscle development (Kim & Hadjiargyrou, 2024). Mustn1 was originally identified as a differentially expressed gene during fracture repair, and is found throughout the musculoskeletal system (Lombardo et al., 2004), as well as being developmentally regulated (Gersch & Hadjiargyrou, 2009; Camarata et al., 2016). More importantly, it has been implicated as a key regulator of myogenesis (Hadjiargyrou, 2018; Kim & Hadjiargyrou, 2024). Notably in mice, Mustn1 is expressed during skeletal muscle development and growth, peaking at 3 months of age (Liu et al., 2010). Additionally, Mustn1 was shown to co‐localize with the satellite cell marker Pax7, reinforcing its potential role in satellite cells and muscle regeneration (Krause et al., 2013). More recently, Ducommun et al. (2024) reported increases in Mustn1 expression in injured gastrocnemius 1 day post‐injury using Mustn1 Knockout (KO)‐first and MEF2C/myogenin promoter–Cre recombinase crossed with β‐actin promoter‐driven Flp recombinase or Actb‐Cre mice. Also, Fu et al. (2025) demonstrated that Mustn1 interacts with Small muscle protein X‐linked (SMPX) and together, they promote myogenic differentiation. In this study, the authors also showed that Mustn1 deficiency delays skeletal muscle regeneration (Fu et al., 2025).

Additional studies from our group using Mustn1 conditional KO mice (Pax7 cre) demonstrated a variety of physiological effects on glucose metabolism, grip strength, gait, peak contractile strength, and myofiber composition (Kim, Singh, Lee, et al., 2023; Kim, Singh, Kaczmarek, et al., 2023). Similarly, a number of other studies reported on the characterization of the Mustn1 promoter (Liu & Hadjiargyrou, 2006; Suarez‐Bregua et al., 2017) as well as during skeletal muscle hypertrophy in many species (reviewed in Kim & Hadjiargyrou, 2024). Lastly, a number of studies also reported on the role of Mustn1 during exercise showing that its expression is upregulated during acute exercise and training (Ducommun et al., 2024; Jensen et al., 2012; Kostek et al., 2007; McKenzie et al., 2011; Oh, 2011; Oh & Oh, 2013).

In this study, our objective was to elucidate whether Mustn1 ablation results in alterations of skeletal muscle regeneration following injury. Using a cardiotoxin (CTX)‐induced muscle injury model (Wang, Lu, & Liu 2022), we investigated muscle regeneration in wild‐type (WT) and Mustn1 KO mice (Pax7‐cre) through functional, histological, and molecular analyses. Specifically, we assessed function, muscle fiber composition, satellite cell dynamics, and gene expression changes across different time points post‐injury. The findings from this study provide additional novel insights into the molecular mechanisms underlying muscle repair and may have implications for therapeutic strategies targeting muscle regeneration disorders.

## 2 METHODS

### 2.1 Skeletal injury model

All animal care and experiments were carried out in accordance with the guidelines of the Institutional Animal Care and Use Committee approved protocols and met or exceeded all federal guidelines for the humane use of animals in research. The animal room was maintained at 21°C, 50% humidity and a 12 h per 12 h light–dark cycle. All mice had access to standard chow and water ad libitum, as well as enrichment material (e.g. nesting squares and plastic housing). The generation and characterization of the Mustn1 KO (Pax7 Cre) mice was previously reported by our laboratory (Kim, Singh, Lee, et al., 2023; Kim, Singh, Kaczmarek, et al., 2023). WT and Mustn1 KO male mice (*n* = 5/genotype, 2 months old) were anesthetized with 3% isoflurane and maintained with 1.5% isoflurane inhalation. Nair was applied to the right leg of each mouse to remove fur, and then the leg to be injected was cleaned with an alcohol prep pad. A 29G needle was used to inject 100 μL of 10 μM CTX, *Naja pallida* (Sigma, #11061‐96‐4) into the Tibialis anterior (TA) muscle of the right leg. We chose the TA because of its accessible spatial location and because it is well known to have a mixture of fiber types. Mice were allowed to recover for several minutes before being returned to their cages.

### 2.2 Ladder climbing

To assess the function of the injured leg, mice (*n* = 5 males/genotype) were timed while climbing a ladder 0.5 meters at an 85° incline. Mice were acclimated to the ladder 1 day before the first assessment. For each mouse, five trials were completed, with a 1‐min rest in between each. Ladder climbing was assessed immediately before CTX injection (control), and on Days 2, 5, and 10 post‐CTX injection. Any trial where a mouse turned around on the ladder was excluded from the analysis. As such, for each mouse we recorded 5 completed trials for each time point.

### 2.3 RNA isolation/cDNA synthesis

Mice (*n* = 3 males/genotype) were sacrificed 2 h (Day 0), Day 2, 5, and 10 following CTX injection. The TA muscles were harvested under sterile conditions post‐mortem. Muscle samples were placed in 1 mL of TRIzol reagent (Invitrogen, #15596026), as per manufacturer's protocol, then homogenized using a motorized tissue homogenizer at 4°C to prevent sample degradation. Samples were stored at −80°C. RNA was extracted according to the manufacturer's protocol and as we previously described (Hadjiargyrou et al., 2016; Tsadaris et al., 2024). Isolated RNA samples were further processed using the RNeasy Mini Kit (Qiagen, #74104) per manufacturer's instructions. The final RNA pellet was eluted with 30–50 μL of RNAse‐free water, and RNA quality was assessed using a Nanodrop spectrophotometer 2000C (Thermo Fisher). RNA samples were stored at −80°C.

cDNA synthesis was performed using the ReadyScript cDNA Synthesis Mix (Sigma‐Aldrich, #RDRT‐100RXN). Thawed RNA and ReadyScript Mix were combined in 0.2 mL microtubes with 4 μL of ReadyScript Mix, RNA, and brought to a final volume of 20 μL with RNAse‐free water. Reactions were vortexed gently, centrifuged briefly, and subjected to the following conditions: 5 min at 25°C, 30 min at 42°C, 5 min at 85°C, with a final hold at 4°C. The cDNA samples were then stored at −20°C.

### 2.4 Quantitative PCR (qPCR)

Quantitative PCR (qPCR) was conducted using SYBR Green PCR master mix (Roche Diagnostics, #04707516001) using a LightCycler 480II (ROCHE). Each 10 μL reaction included 5 μL of 2X SYBR Green PCR master mix, 0.5 μL each of forward and reverse primers, 3 μL of RNAse‐free water, and 1 μL of cDNA. Primer sequences and amplicon sizes were previously used and reported by our laboratory (Kim, Singh, Kaczmarek, et al., 2023). Thermocycler conditions were set as follows: initial denaturation at 95°C for 5 min, followed by 45 cycles of 95°C for 20 s, annealing for 35 s at 61°C, and extension at 72°C for 35 s for 50 cycles. Each gene was tested in quadruplicate (*n* = 4) to ensure reproducibility and accuracy of the results. Fold change in expression was quantified for each time point by dividing the concentration of the target gene by the concentration of the 18S rRNA housekeeping gene using their own standard curves for each PCR run.

### 2.5 Histology

Mice (*n* = 2–3 males/genotype) were sacrificed on either 2 h (Day 0), Day 2, 5, and 10 following CTX injection. The lower hind legs of the mice were harvested and embedded in OCT, frozen in liquid nitrogen, and sectioned at 8 μM. Sections were then stained with hematoxylin and eosin (H & E) using a standard protocol and as previously described by our laboratory (Kim, Singh, Lee, et al., 2023).

### 2.6 Fiber typing immunofluorescence

Muscle fiber typing was performed using the protocol from Bloemberg and Quadrilatero (2012). Three slides (2–3 muscle sections/slide) per leg (*n* = 2–3 mice) were blocked for 1 h with 10% goat serum in PBS. Primary antibodies were incubated overnight at 4°, followed by secondary antibodies incubated for 1 h at room temperature (RT). Slides were mounted using Invitrogen mounting medium with DAPI (Thermo Fisher, #62248). Primary antibodies and dilutions were as follows: MHCI (BA‐F8, 1:50), MHCIIa (Sc‐71, 1:500), MHCIIb (BF‐F3, 1:100). All antibodies were purchased from Developmental Studies Hybridoma Bank. Goat anti‐mouse secondary antibodies (IGM‐488, #A21042; IgG1‐568, #A21124; IgG2‐647, #A21242) were purchased from Invitrogen and were used at 1:250. We did not use an antibody to identify type Iix fibers; they remained unstained. Slides from two mice with three muscle sections each from the injected leg and three from the non‐injected leg (control) were imaged using an Axio Scan.Z1 (Carl Zeiss Microscopy) microscope and were analyzed. Lastly, fiber types were quantified using pixel classification in Qupath.

The protocol for Pax7 immunofluorescence staining was adopted by Wang et al. (2023). Briefly, three slides (2–3 muscle sections/slide) were fixed with 10% formalin for 15 minutes and permeabilized at RT with 0.2% Triton X‐100 for 10 min. The slides were then blocked in 10% goat serum for 1 h followed by incubation with primary antibody (1:50, Developmental Studies Hybridoma Bank, #AB_528428) overnight at 4°C and secondary antibody (Invitrogen, #A21242) for 1 h at RT. Slides were mounted using Invitrogen mounting medium with DAPI (Thermo Fisher, #62248). Pax7 positive cells were identified via object classification in Qupath.

### 2.7 Cross‐sectional area and centrally nucleated fiber measurements

To measure muscle cross‐sectional area, we used the fluorescently labeled individual muscle fibers in Qupath. Specifically, Type I, IIa, and IIb fibers were selected as annotations using the wand tool and their areas were measured. Three histological sections from each animal (*n* = 2/group) were used for control, Day 0, 2, 5, and 10 from both WT and Mustn1 KO mice.

Lastly, centrally nucleated fibers were identified using H & E stained muscle sections. Using the grid feature in Qupath to segment the muscle into several sections, fibers with centrally located nuclei were manually counted (*n* = 1–3 sections per animal, from each animal [*n* = 2–3/group] for both WT and Mustn1 KO mice). Central nucleation counts were averaged within each animal and reported as individual samples. One section each, from five animals, was used for control for both strains.

### 2.8 Statistical analyses

All data are presented as dot plots, with the median represented as the central line and whiskers comprising the 95th confidence intervals. The data were assessed for normality using Shapiro–Wilk tests and not all passed. Accordingly, a mixed‐effects, model was used to compare time and genotype, with sphericity not assumed. When significant effects were found, the identify of differences were determined using Tukey's multiple comparisons tests. All analyses were performed at an alpha level of 0.95 using Prism (Version 10.5.0, GraphPad).

## 3 RESULTS

### 3.1 Skeletal muscle morphology

**FIGURE 1:**
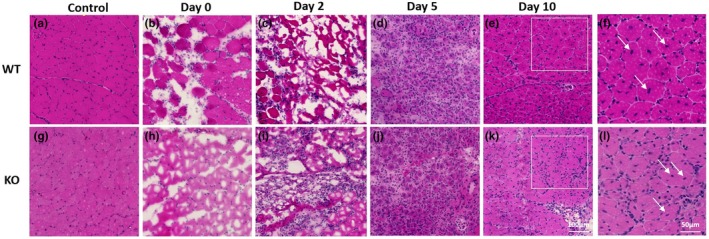
Histological analyses of tibialis anterior muscle. Representative images of WT (a–f) and KO (g–l) tibialis anterior muscle stained with H & E. Control (uninjured) and 0, 2, 5, and 10 days post‐injury. f and l are magnified regions depicted in e and k, respectively. Arrows indicate fibers with centrally located nuclei. Scale bar = 100 μm and 50 μm.

Staining of TA muscle with H & E pre‐ and post‐injection at Days 0, 2, 5 and 10 did not reveal any major morphological differences between the WT and Mustn1 KO mice (Figure 1). Pre‐injection (Control) skeletal muscle looks intact as expected in both mouse strains (Figure 1a,g), but as soon as 2 h post‐injection (Day 0), we can clearly see fiber degeneration (Figure 1b,h) which continues till Day 2 (Figure 1c,i). By Day 5 (Figure 1d,j) and 10 (Figure 1e,k), we can detect regeneration of the muscle fibers with lots of centrally located nuclei, which are also seen at higher magnification from the Day 10 samples and indicated by the arrows (Figure 1f,l).

### 3.2 Muscle cross‐sectional area and centrally nucleated fibers

**FIGURE 2:**
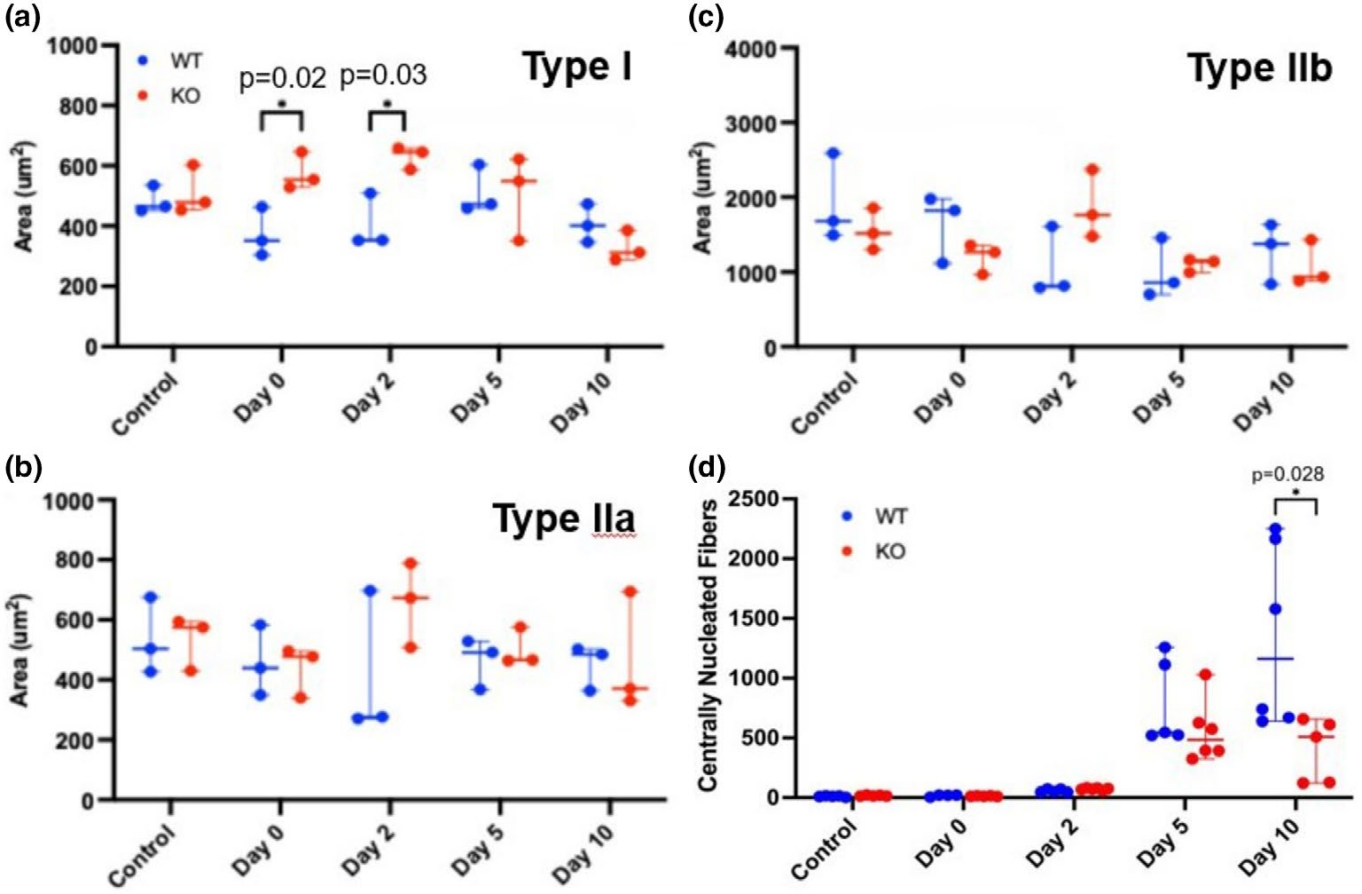
Muscle cross sectional area and central nucleated fibers. Muscle cross sectional area comparison between WT and Mustn1 KO mice for Type I (a), Type IIa (b) and Type Iib (c) fiber. (d) Centrally nucleated fibers WT and Mustn1 KO mice. Comparisons were made using a mixed‐effects model followed post‐hoc Tukey's tests to identify pairwise differences between genotypes and times.

As a function of regeneration metrics, we also measured fiber cross‐sectional area and centrally nucleated fibers. Examination of Type I fiber cross‐sectional area revealed changes between the WT and Mustn1 KO mice at Day 0 and 2. Specifically, at both Day 0 and 2, there is an increase in the cross‐sectional area of Type I fibers in the Mustn1 KO mice (35.3% and 35.8%, respectively) as compared to the WT (Figure 2a). No statistically significant changes were observed between the two strains of mice in the cross‐sectional area of Type IIa or Type IIb fibers at any time points (Figure 2b,c). When we examined centrally nucleated fibers, a statistically significant difference was observed at Day 10, with the WT showing double the number in comparison to the Mustn1 KO mice (Figure 2d).

### 3.3 Muscle fiber types

**FIGURE 3:**
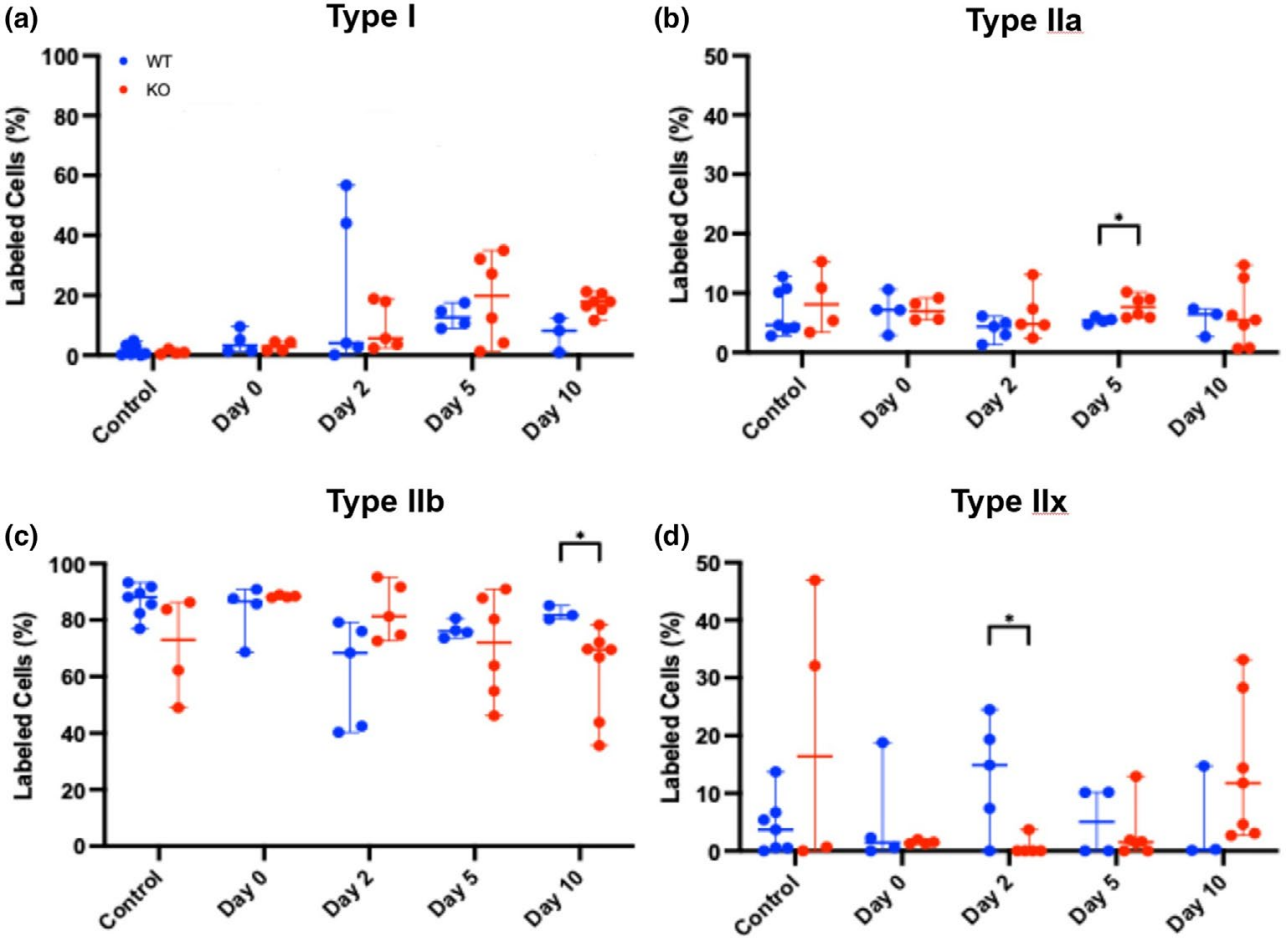
Fiber typing: Quantitative data. (a) Type I fibers; (b) Type IIa fibers; (c) Type IIb fibers; (d) Type IIx fibers. Control refers to uninjured and Day 0–10 refer to post‐injured samples. All *p*‐values were calculated using a Tukey's multiple comparisons test.

**FIGURE 4:**
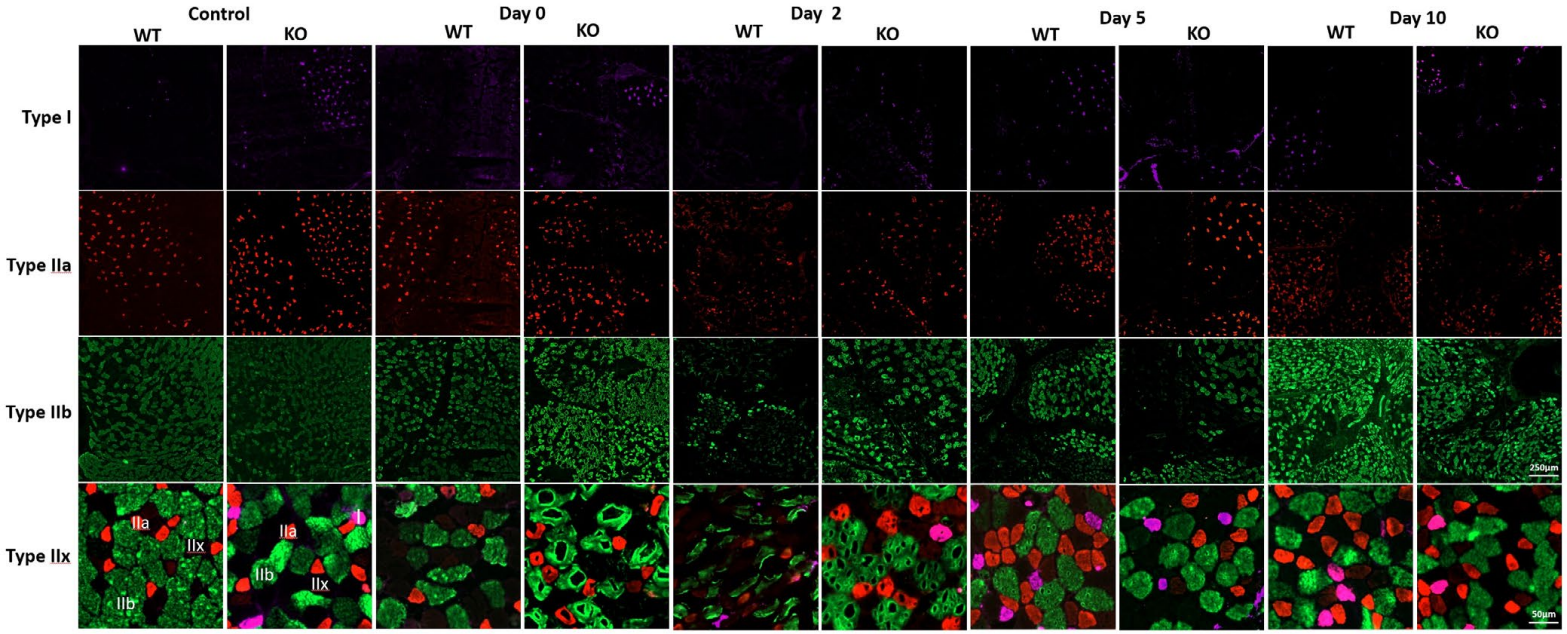
Fiber typing: Qualitative data. Representative images show the various fiber types from each strain and time point. Control refers to uninjured and day 0–10 refer to post‐injured samples. For the Type Iix fiber, the images appear at a higher magnification so that each distinct fiber is shown: Type I (pink), Type IIa (red), Type IIb (green), and Type Iix (black). Scale bars = 250 and 50 μm.

To examine whether there were any structural differences in muscle fiber types (Type I, IIa, IIb, and IIx) during regeneration between the two mouse strains, we employed immunofluorescent analyses of the TA before and after CTX injection. Examination of Type I fibers indicates no statistically significant difference between the two strains at all time points, but there was a higher trend at post‐injury Day 10 with a ~142% increase in the Mustn1 KO mice (Figure 3a). This is reflected in the qualitative data as well (Figure 4). With Type IIa fibers, we also detected a statistically significant difference; Mustn1 KO mice display a ~42% increase in comparison to those seen with WT mice (Figures 3b and 4) at Day 5 post‐injury. The other time points were insignificant between the two mouse strains. In contrast, with fibers Type IIb and IIx, we found the opposite; the WT mice show a statistically higher percentage of Type IIb (~25%) at post‐injury Day 10 (Figures 3c and 4) and a massive increase (~1783%) in Type IIx at post‐injury Day 2 (Figures 3d and 4). The other time points were insignificant between the two mouse strains.

### 3.4 Pax 7^+^ cells

**FIGURE 5:**
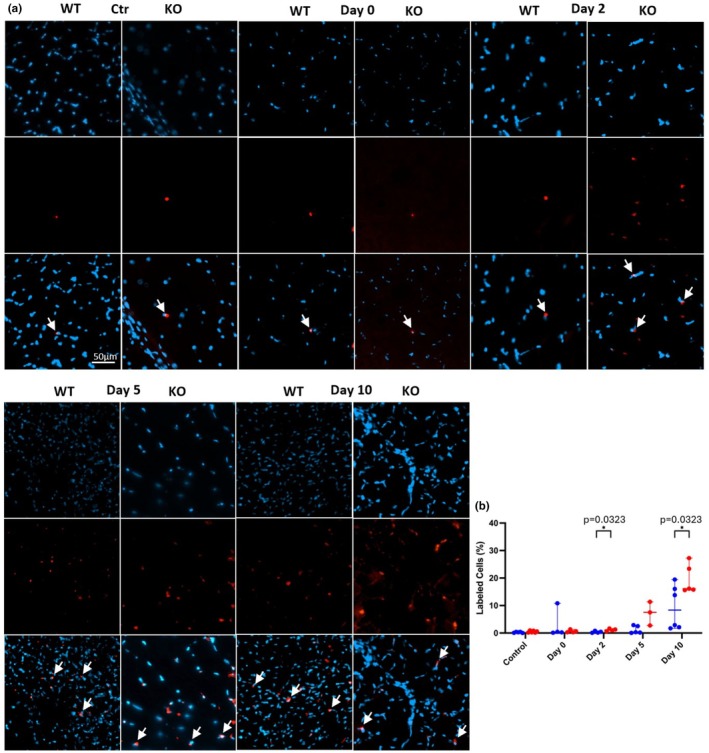
Pax 7 expression. (a) Representative images show Pax7^+^ fibers from each strain and time point. Control refers to uninjured and Day 0–10 refer to post‐injured samples. (b) Graph indicates the quantitative data All *p*‐values were calculated using a Tukey's multiple comparisons test. Scale bar = 50 μm.

We also wanted to monitor Pax7 expression since (a) the Mustn1 genetic modification targeted satellite cells; (b) these cells play a key role in skeletal muscle regeneration; and (c) Pax7 can serve as an indicator of myogenic impairment. Statistically significant differences were observed at post‐injury days 2 and 10 between the two strains of mice, with the Mustn1 KO showing greater numbers of Pax7^+^ cells (4× and 2× more at Day 2 and 10, respectively; Figure 5a,b). We also observed a trend with more (~3×) Pax7^+^ cells at post‐injury Day 5 in the Mustn1 KO mice, but the data was not significant (Figure 5a,b).

### 3.5 Myogenic expression

**FIGURE 6:**
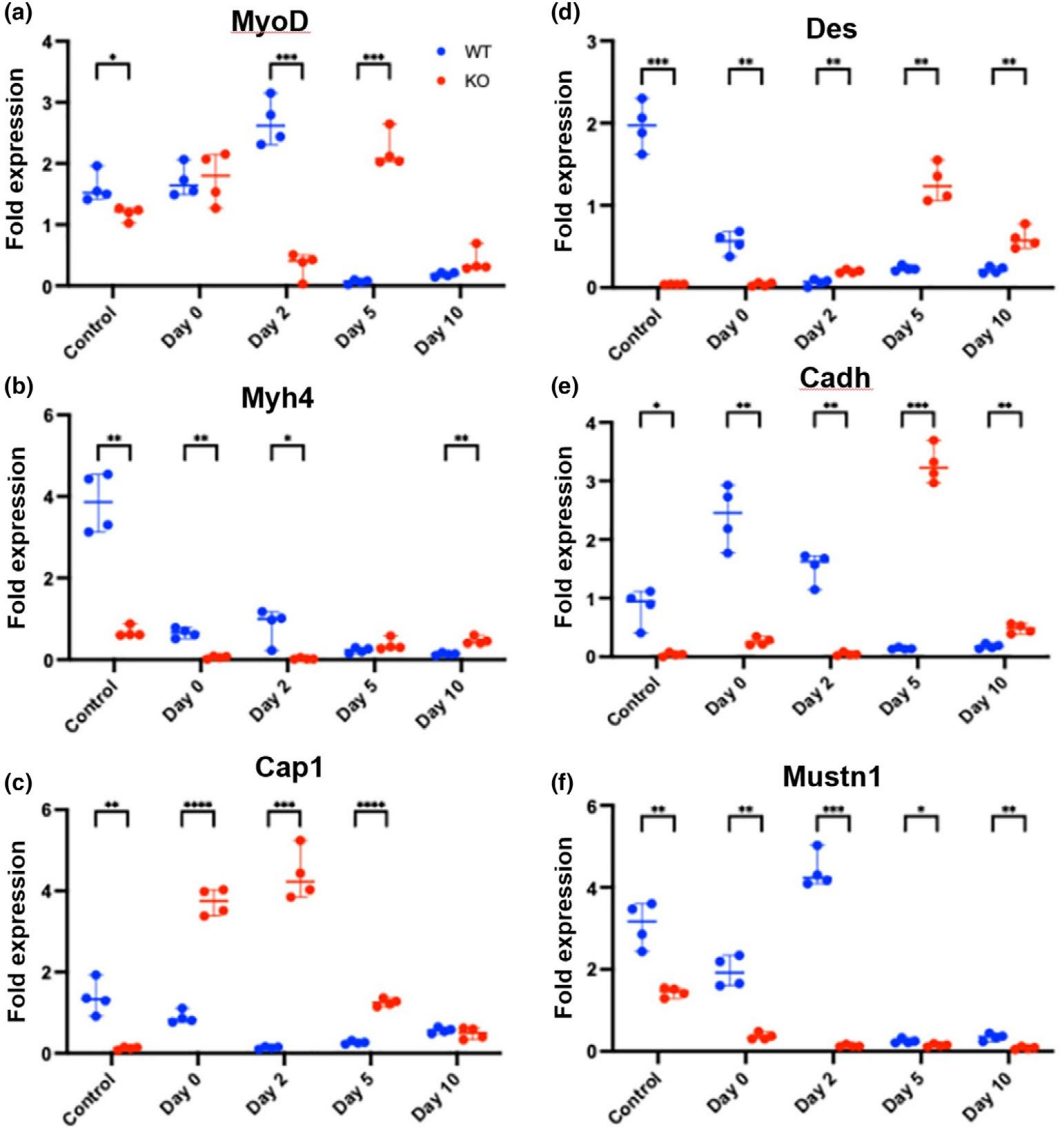
Gene expression analyses. Expression levels of myogenic marker genes (a) MyoD, (b) Myh4, (c) Cap1, (d) Des, (e) Cadh, and (f) Mustn1. Control refers to uninjured and Day 0–10 refer to post‐injured samples. All *p*‐values were calculated using a Tukey's multiple comparisons test.

We also investigated whether there were any differences in the expression of skeletal muscle marker genes between the two strains of mice. As such, we investigated mRNA expression levels of the differentiation, MyoD, Myh4, and Cap1, as well as the mature muscle markers, Des, Cadh. Lastly, we also monitored the expression of Mustn1. The expression of MyoD was higher in the WT mice versus Mustn1 KO mice in the Control (~1.35 fold) as well as post‐injury Day 2 (~7.8 fold) (Figure 6a). In contrast, MyoD expression was higher in the Mustn1 KO mice at day 5 (~36.7 fold) (Figure 6a). Similarly, the expression of Myh4 was higher in the WT mice versus Mustn1 KO mice in the Control (~5.67 fold) as well as Day 0 (~11.4 fold) and post‐injury Day 2 (~42 fold) (Figure 6b). In contrast, Myh4 expression was statistically higher in the Mustn1 KO mice at 10 (~0.33 fold) (Figure 6b). Although there was a higher trend with the Mustn1 KO mice at Day 5, the difference was not significant (Figure 6b). For Cap1, we only detected a higher fold (~11.4 fold) change of expression in the WT versus Mustn1 KO mice in the control samples and no significant differences at Day 10 (Figure 6c). But for Day 0, 2 and 5, we observed significantly higher levels of expression in the Mustn1 KO mice with ~4.2, 43.8 and 6.0 fold, respectively (Figure 6c).

With Des, we detected significant increased expression levels in the WT mice with the Control (~49 fold) and Day 0 (~13.5 fold) samples but the reverse at Days 2 (~3.2 fold), 5 (~5.47 fold), and 10 (~3 fold) in the Mustn1 KO mice (Figure 6d). Cadh expression was significantly increased in WT mice in the Control (~21.2 fold), Day 0 (~9.2 fold), and Day 2 (~38 fold) samples, but decreased at day 5 (~23.3 fold) and 10 (~2.7 fold) (Figure 6e). Lastly, as expected, Mustn1 was significantly decreased at every time point in the Mustn1 KO mice (Figure 6f). The low level of expression detected in the control sample may be due to the possible presence of tendon left in the muscle, which is also known to express Mustn1 (Lombardo et al., 2004; Janton et al., 2025). It is also interesting to note that Mustn1 expression in the WT appears to be at the highest levels at post‐injury Day 2 (Figure 6f), which coincides with satellite cell activation and proliferation (Wang, Lu, & Liu 2022).

### 3.6 Functional test

**FIGURE 7:**
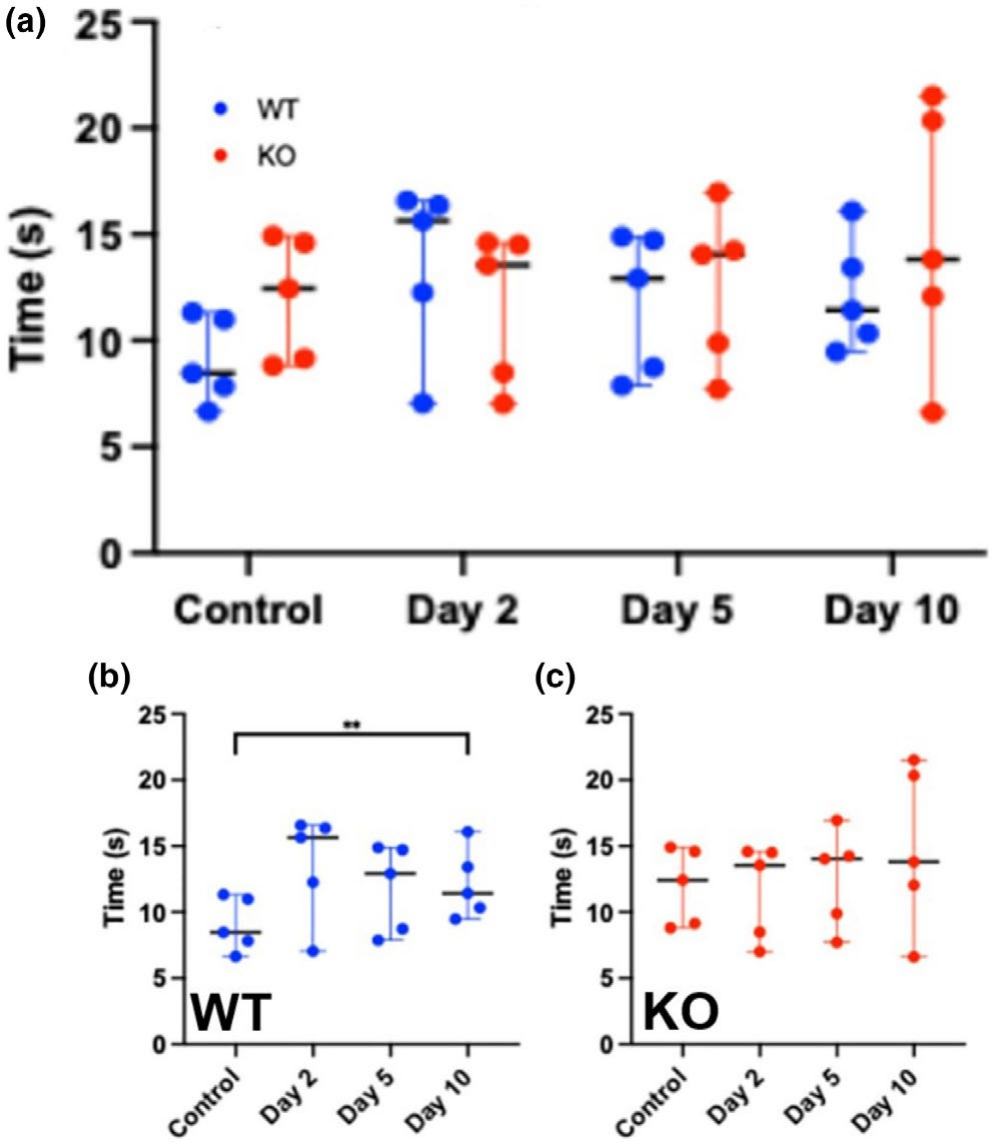
Ladder climbing. (a) Ladder climbing comparison between WT and Mustn1 KO mice (*n* = 5/strain); (b) WT mice ladder climbing; (c) Mustn1 KO mice ladder climbing. All *p*‐values were calculated using a Tukey's multiple comparisons test tests between genotypes as well as per time point.

In order to assess whether any of the aforementioned changes resulted in differences in skeletal muscle function following injury in the WT and Mustn1 KO mice, we utilized an 85‐degree incline ladder climbing. A change in climbing gait was observed in both strains of mice. While climbing, the mice tended to abduct the injured leg, carrying it widely to take steps on the ladder (data not shown). There was no statistically significant difference between the two strains of mice at any time point (Figure 7a). Additionally, the WT mice climbed 50% slower on the second day after injury than pre‐injection (Control) (Figure 7b), and their climbing time decreased on Days 5 and 10 but did not return to the pre‐injection time. Only Day 10 represents a statistically significant difference from control and shows a ~35% decrease in climbing time. The Mustn1 KO mice did not show any statistically significant difference in climbing time between control and after injury (Day 2, 5, and 10, Figure 7c).

## 4 DISCUSSION

In this study, we examined the effects of Mustn1 deletion on skeletal muscle regeneration by analyzing morphological changes, fiber‐type composition, myogenic gene expression, and functional performance. Our data suggest that although Mustn1 deletion does not grossly impair the muscle's ability to regenerate after injury (CTX injection), it appears to influence the composition of regenerating fibers and the temporal expression of myogenic marker genes. Collectively, these results indicate that Mustn1 has a subtle but potentially significant regulatory role in skeletal muscle regeneration.

This is not surprising, since we previously reported that Mustn1 deletion affects glucose metabolism, grip strength, gait, peak contractile strength, and myofiber composition (Kim, Singh, Lee, et al., 2023; Kim, Singh, Kaczmarek, et al., 2023). More recently, Fu et al. (2025) also reported that Mustn1 KO mice exhibit reduced muscle mass and fiber cross‐sectional area as well as decreased exercise endurance and delayed muscle regeneration. We also showed using transgenic mice (GFP expression driven by the Mustn1 promoter (Liu & Hadjiargyrou, 2006)) that Mustn1 plays a role in the development of skeletal muscle as well as during regeneration, peaking at 3 days post‐injury with decreasing levels observed at 5 and 10 days (Krause et al., 2013), consistent with our results herein. Finally, Ducommun et al. (2024), also reported increases in Mustn1 expression in injured gastrocnemius 1 day post‐injury using Mustn1 KO‐first and MEF2C/myogenin promoter–Cre recombinase crossed with β‐actin promoter‐driven Flp recombinase or Actb‐Cre mice. Despite these data, we did not observe any gross defects in the overall morphology of regenerating fibers between WT and Mustn1 KO mice. Both strains exhibited fiber degeneration shortly after injury (Day 2) and regeneration by Day 5 and 10, characterized by centrally located nuclei. Although we did not detect any differences in the numbers of centrally nucleated fibers between the two mouse strains, significant temporal differences were observed within each strain. More importantly, we did observe that Mustn1 KO mice exhibited higher proportions of Type I (trend) and Type IIa (significant) fibers at later stages of regeneration, whereas WT mice retained higher percentages of Type IIb and IIx fibers. Also, the Mustn1 KO mice displayed significant increases in Type I fiber area at Day 0 and Day 2 following CTX injection. This increase in cross‐sectional area of Type I fibers in Mustn1 KO mice (35.3% and 35.8%, respectively) may suggest a potential compensatory mechanism or altered regenerative response in the absence of Mustn1. Prior research has established that fast‐twitch fibers (IIb and IIx) are more susceptible to injury but regenerate rapidly under normal conditions (Schiaffino & Reggiani, 2011). The shift toward a higher percentage of oxidative fibers (Type IIa) in the KO mice suggests that Mustn1 may play a role in maintaining or restoring the fast‐twitch phenotype after injury. This is consistent with findings from previous studies showing that Mustn1 is upregulated during periods of skeletal muscle hypertrophy in many vertebrate species (Fu et al., 2025; Gu et al., 2024; Li et al., 2020; Liu et al., 2010; Rong et al., 2024; Wang, Liang, et al., 2022; Xu et al., 2015; Yu et al., 2023; Zhu et al., 2021).

In vitro data lend additional credence to the importance of Mustn1 in skeletal muscle. For example, we previously reported that Mustn1 plays a role in myogenic differentiation, especially myofusion and myotube formation (Liu et al., 2010), critical processes for regenerating muscle fibers. Additionally, it was reported that Mustn1 expression increased in conjunction with the proliferation of chicken skeletal muscle satellite cells (SMSCs) and its knockdown results in downregulation of Pax7, Cdk‐2, MyoD, MyoG, MyHC, and MyH1B (Hu et al., 2021). Moreover, cultured skeletal muscle precursor cells isolated from Mustn1 KO mice displayed inhibited proliferation, migration, myofusion, and differentiation (Fu et al., 2025).

Our data also demonstrate that Mustn1 deletion alters the expression pattern of key myogenic markers during skeletal muscle regeneration. Specifically, we observed that WT mice generally expressed higher levels of MyoD, Myh4, Des, and Cadh early post‐injury, consistent with Mustn1's role in inducing myoblast differentiation and fusion (Fu et al., 2025; Hu et al., 2021; Liu et al., 2010). The delayed expression of these markers in the Mustn1 KO mice at the early time points (Day 0–2), followed by recovery at later days (Fu et al., 2025; Gersch et al., 2012; Gersch & Hadjiargyrou, 2009; Gu et al., 2024; Hadjiargyrou, 2018; Hadjiargyrou et al., 2016), suggests that Mustn1 may be important for the temporal activation of myogenic programming (i.e., signaling pathways and other key cellular processes required for tissue repair), especially in satellite cells. This temporal dysregulation could be a contributing factor to the altered fiber‐type profile we observed and may partially explain the lack of serious functional impairment, as muscle regeneration ultimately proceeds but with a slightly different fiber type composition. Downregulation of these and other myogenic differentiation marker genes were also reported in the studies of Hu et al. (2021) and Fu et al. (2025). These results suggest that Mustn1 is not absolutely required for the initiation of muscle regeneration but may influence the process.

Our functional test, ladder‐climbing, revealed no significant differences between WT and KO mice post‐injury, consistent with previous reports that showed that functional deficits can be subtle in skeletal muscle (Issa et al., 2012; Larrinaga et al., 2024; Percival et al., 2008). Noteworthy is the modest trend toward slower functional recovery in WT mice compared to KO mice at earlier time points, and with WT mice showing significant recovery only by Day 10. This may reflect differences in fiber‐type regeneration as indicated by our data and is consistent with previous data showing that Mustn1 knockdown can delay but not completely block myoblast differentiation in vitro (Liu et al., 2010; Hu et al., 2021), suggesting that other compensatory pathways may lessen functional loss in vivo.

We have also identified some limitations of our study which include: (1) a small “*n*” number; (2) only use of male mice; (3) a relatively short follow‐up period (up to 10 days post‐injection); (4) functional testing was performed on all four limbs; and (5) the use of only a single muscle injury model (TA). Regardless, this study expands on previous work by demonstrating that Mustn1 deletion alters muscle fiber‐type composition and myogenic gene expression in vivo without causing gross defects in regeneration. Further, our data indicate that Mustn1 acts as a modulator rather than an indispensable factor for muscle regeneration. This is consistent with earlier work that described its role as a regulator of chondrogenesis (Gersch et al., 2012; Gersch & Hadjiargyrou, 2009) and myogenesis (Ducommun et al., 2024; Fu et al., 2025; Hu et al., 2021; Kim, Singh, Lee, et al., 2023; Kim, Singh, Kaczmarek, et al., 2023) but not as an essential structural gene. In fact, Ducommun et al. (2024) reported that Mustn1 is a secreted microprotein that may influence extracellular composition based on proteomics analysis of muscle from Mustn1‐deficient mice that revealed differences in extracellular matrix composition. More recently, we reported on robust mural cell‐state colocalization of Mustn1 and Acta2 (encoding alpha smooth muscle actin, aSMA) within blood vessels of skeletal muscle (Janton et al., 2025). As skeletal muscle regeneration involves significant angiogenesis and extracellular remodeling, it may be conceivable that Mustn1, as a secreted protein, may play a role in these processes.

Compared to the many studies that reported on Mustn1 in skeletal muscle (Kim & Hadjiargyrou, 2024) and beyond those cited herein, these findings add to our understanding of how regulatory genes like Mustn1 may shape the regenerative capacity of skeletal muscle by fine‐tuning or modulating the process. Additionally, previous studies have focused on core transcription factors such as Pax7, MyoD, and Myf5 (Hernández‐Hernández et al., 2017; Zammit, 2017), but fewer have investigated the contribution of modulatory factors that may affect the timing and fine‐tuning of regeneration, especially the development and switching of fiber types. Our results suggest that Mustn1 is such a factor, and its loss may affect regeneration toward a more oxidative, fatigue‐resistant phenotype. Additional research can determine whether targeting Mustn1 can have therapeutic implications in situations such as muscular dystrophies or sarcopenia where enhanced oxidative capacity is necessary.

Finally, as the mice we used in this study were young (2 months old), our results may have clinical implications for the treatment of pediatric muscle disorders (i.e., muscular dystrophy) where regeneration is impaired. Although there is no definitive data indicating the functional significance of Mustn1 in muscular dystrophies, it is certainly possible given the existing data by our laboratory as well as indirect evidence by others. For example, Balagopal et al. (2006), reported that oxandrolone treatment in four children with Duchenne Muscular Dystrophy (DMD), enhanced MHC synthesis, subsequently reducing muscle degeneration. More importantly, Mustn1 was among the downregulated genes in DMD muscles in response to oxandrolone. In an experiment using a canine model of Golden Retriever Muscular Dystrophy (GRMD) Robriquet et al. (2015) reported that Mustn1 was one of the upregulated following transplantion of skeletal muscle‐resident stem cells leading to an enhancement in skeletal muscle fiber regeneration. Collectively, these results implicate Mustn1 involvement in such pediatric skeletal muscle disorders like muscular dystrophies.

## AUTHOR CONTRIBUTIONS

**Kaitlin Tagliaferri**: data curation; formal analysis; methodology; writing—review and editing. **Nithya Thomas**: data curation; formal analysis; methodology; writing. **Michael Atallah**: data curation. **Stavroula Triantafillou**: data curation. **Michael Hadjiargyrou**: conceptualization; data curation; formal analysis; funding acquisition; investigation; methodology; project administration; resources; supervision; validation; visualization; original draft; writing—review and editing.

## FUNDING INFORMATION

Research reported in this publication was supported by grant R15HD092931 (Michael Hadjiargyrou), from the Eunice Kennedy Shriver National Institute of Child Health & Human Development of the National Institutes of Health, and an Institutional Support for Research and Creativity (ISRC) Grant from NYIT (Michael Hadjiargyrou).

## ETHICS STATEMENT

All animal experiments were approved by the Institutional Animal Care and Use Committee (IACUC) of the New York Institute of Technology.
